# Impact of the cerebrospinal fluid-mask algorithm on the diagnostic performance of ^123^I-Ioflupane SPECT: an investigation of parkinsonian syndromes

**DOI:** 10.1186/s13550-019-0558-x

**Published:** 2019-09-03

**Authors:** Yu Iwabuchi, Tadaki Nakahara, Masashi Kameyama, Yohji Matsusaka, Yasuhiro Minami, Daisuke Ito, Hajime Tabuchi, Yoshitake Yamada, Masahiro Jinzaki

**Affiliations:** 1 Department of Radiology, Keio University School of Medicine, 35 Shinanomachi, Shinjyuku-ku, Tokyo, 160-8582 Japan; 2 Department of Diagnostic Radiology, Tokyo Metropolitan Geriatric Hospital and Institute of Gerontology, 35-2 Sakaecho, Itabashi-ku, Tokyo, 173-0015 Japan; 3 Department of Neurology, Keio University School of Medicine, Tokyo, Japan; 4 Department of Neuropsychiatry, Keio University School of Medicine, 35 Shinanomachi, Shinjyuku-ku, Tokyo, 160-8582 Japan

**Keywords:** ^123^I-Ioflupane, ^123^I-FP-CIT, DAT SPECT, Southampton method, Specific binding ratio, CSF-mask

## Abstract

**Background:**

A cerebrospinal fluid (CSF)-mask algorithm has been developed to reduce the adverse influence of CSF-low-counts on the diagnostic utility of the specific binding ratio (SBR) index calculated with Southampton method. We assessed the effect of the CSF-mask algorithm on the diagnostic performance of the SBR index for parkinsonian syndromes (PS), including Parkinson’s disease, and the influence of cerebral ventricle dilatation on the CSF-mask algorithm.

**Methods:**

We enrolled 163 and 158 patients with and without PS, respectively. Both the conventional SBR (non-CSF-mask) and SBR corrected with the CSF-mask algorithm (CSF-mask) were calculated from ^123^I-Ioflupane single-photon emission computed tomography (SPECT) images of these patients. We compared the diagnostic performance of the corresponding indices and evaluated whether the effect of the CSF-mask algorithm varied according to the extent of ventricle dilatation, as assessed with the Evans index (EI). A receiver-operating characteristics (ROC) analysis was used for statistical analyses.

**Results:**

ROC analyses demonstrated that the CSF-mask algorithm performed better than the non-CSF-mask (no correction, area under the curve [AUC] = 0.917 [95% confidence interval (CI) 0.887–0.947] vs. 0.895 [95% CI 0.861–0.929], *p* < 0.001; attenuation correction, AUC = 0.930 [95% CI 0.902–0.957] vs. 0.903 [95% CI 0.870–0.936], *p* < 0.001). When not corrected for attenuation, no significant difference in the AUC was observed in the low EI group between the non-CSF-mask and CSF-mask algorithms (0.927 [95% CI 0.877–0.978] vs. 0.942 [95% CI 0.898–0.986], *p* = 0.11); in the middle and high EI groups, the CSF-mask algorithm performed better than the non-CSF-mask algorithm (middle EI group, AUC = 0.894 [95% CI 0.825–0.963] vs. 0.872 [95% CI 0.798–0.947], *p* < 0.05; high EI group, AUC = 0.931 [95% CI 0.883–0.978] vs. 0.900 [95% CI 0.840–0.961], *p* < 0.01). When corrected for attenuation, significant differences in the AUC were observed in all three EI groups (low EI group, AUC = 0.961 [95% CI 0.924–0.998] vs. 0.942 [95% CI 0.895–0.988], *p* < 0.05; middle EI group, AUC = 0.905 [95% CI 0.843–0.968] vs. 0.872 [95% CI 0.800–0.944], *p* < 0.005; high EI group, AUC = 0.954 [95% CI 0.917–0.991] vs. 0.917 [95% CI 0.862–0.973], *p* < 0.005).

**Conclusion:**

The CSF-mask algorithm improved the performance of the SBR index in informing the diagnosis of PS, especially in cases with ventricle dilatation.

## Background

Dopamine transporter (DAT) single-photon emission computed tomography (SPECT) is an imaging modality that can effectively differentiate neurodegenerative parkinsonian syndromes (PS), including Parkinson’s disease (PD) and dementia with Lewy bodies (DLB) from other neurological disorders not characterized by dopaminergic degeneration, such as Alzheimer disease, drug-induced Parkinsonism, and essential tremor [1, 2]. However, a previous study indicated that a suboptimal inter-observer agreement may lead to variable interpretation of DAT SPECT images, indicating that the efficacy of DAT SPECT may rely on visual interpretation [3]. Quantitative assessments are therefore used in addition to visual interpretation when performing DAT SPECT, and previous reports have indicated that a combination of visual interpretation and quantitative assessment achieves more accurate diagnoses [4–6]. Quantitative assessments, such as the specific binding ratio (SBR), are particularly effective in cases with subtle reductions in striatal tracer uptake, which are difficult to register with visual interpretation alone.

Tossici-Bolt et al. developed the Southampton method, a semi-quantitative method based on the volume of interest (VOI), that has gained widespread use. Specifically, the method applies a large pentagonal prism-shaped VOI setting that encompasses a wide area around the striatum [7], thereby reducing the partial-volume effect. This method defines the SBR index as the count concentration of the striatal VOI (reflecting specific binding) divided by the count concentration of the whole brain except for the striatum (reflecting non-specific binding). Although this method reduces the harmful influence of the partial-volume effect and inter-operator variability [7], it has some disadvantages. One disadvantage is that the striatal VOI cannot be divided into the caudate nucleus and the putamen; thus, the diagnostic performance is not superior compared to the VOI settings where the striatal VOI is divided. Another disadvantage is that it is marred by SBR index fluctuations in cases of brain atrophy or cerebral ventricle dilatation because the low-count areas caused by cerebrospinal fluid (CSF) have negative influences on both the striatal and reference VOI counts [8–10]. Also, Furuta et al. previously demonstrated the impact of ventricular enlargement on the SBR index with a three-dimensional (3D)-striatum digital brain phantom [10].

Recently, the CSF-mask algorithm has been developed to reduce the aforementioned influence of CSF-low-counts [8]. Camicioli et al. reported that ventricular dilatation occurs early in the course of significant cognitive decline in patients with PD, and possibly reflect losses of both gray and white matter. Therefore, the CSF-mask algorithm is expected to be useful when calculating an SBR index in such cases [11]. The purpose of this study was to assess the impact of the CSF-mask algorithm on the diagnostic accuracy of the SBR index for PS, and to examine whether the effect of the CSF-mask algorithm differed depending on the extent of cerebral ventricular dilatation; indeed, we hypothesized that the more brain atrophy progresses, the better the effect of the CSF-mask algorithm. To the best of our knowledge, no prior clinical reports have assessed the performance of the CSF-mask algorithm in diagnosing PS.

## Materials and methods

### Patients

**Fig. 1:**
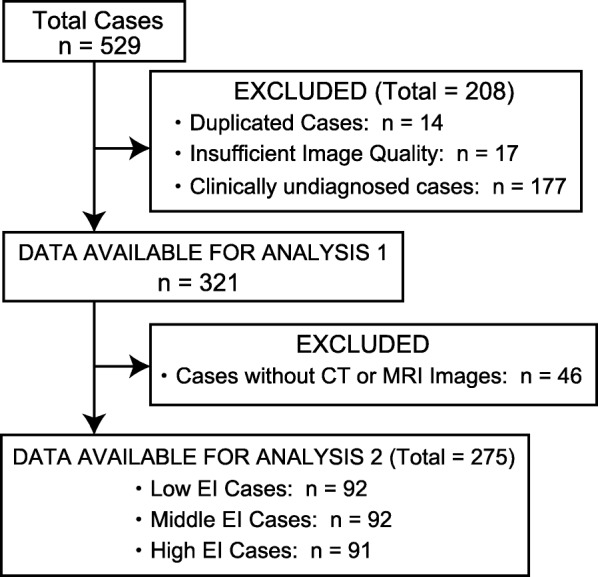
Flow diagram of patient enrollment, eligibility, and exclusion criteria of the dataset. *CT* computed tomography, *MRI* magnetic resonance imaging, *EI* Evans index

This single-center retrospective study included 529 consecutive patients who underwent DAT SPECT from February 2014 to May 2017. Of these patients, 208 were excluded from the study on account of having duplicated or clinically undiagnosed cases (*n* = 14 and 177, respectively) or insufficient image quality (*n* = 17), which included cases with cerebral hemorrhage or brain infarction. Concerning the 177 patients who were clinically undiagnosed cases, they did not meet the diagnostic criteria for any disease finally. In addition, some patients who were difficult to follow due to hospital change were also excluded. Of the remaining 321 patients (median age, 68.9 years; range, 17–91 years; men/women, 176/145), 163 with PS and 158 without PS (NPS) were included in the assessment of the accuracy of the SBR index for diagnosing PS (analysis 1). Of the 163 patients with PS, 114 were diagnosed with PD based on the clinical diagnostic criteria of the UK Parkinson’s disease society brain bank [12]; the remaining 49 patients were diagnosed with atypical PS, including clinical DLB (*n* = 22), multiple system atrophy (MSA) (*n* = 11), and progressive supranuclear palsy (*n* = 16), on the basis of established diagnostic criteria [13–15]. Of the 11 patients with MSA, eight were clinically diagnosed with MSA-Parkinsonism and three were diagnosed with MSA-Cerebellar. Patients with essential tremor, drug-induced Parkinsonism, and normal pressure hydrocephalus were included in the NPS group. In addition, the NPS group included patients whose symptoms, such as tremor, improved during follow-up and were not clinically diagnosed with a degenerative disease. Of the 321 patients, 275 who had CT or MRI images were included in the evaluation of the impact of ventricular dilatation on the CSF-mask algorithm (analysis 2). Based on the Evans index (EI) calculated with CT or MRI images, we divided the enrolled cases into three groups according to the extent of ventricular dilatation: low, middle, and high EI groups (*n* = 92, 92, and 91, respectively). A flow diagram presents the number of participants at each stage of the study (Fig. 1).

The institutional review board of Keio University School of Medicine granted permission for this retrospective review of imaging and clinical data and waived the requirement for obtaining informed consent from the patients (approval number: 20150441).

### SPECT acquisition and reconstruction

Using the Discovery NM/CT 670 or Discovery NM 630 (GE Healthcare, Milwaukee, WI) mounted with a FAN beam collimator, SPECT images were acquired 3 h after the injection of ^123^I-Ioflupane (185 MBq). The imaging parameters were as follows: matrix size, 128 × 128; pixel size, 4.4 mm; slice thickness, 4.4 mm; and energy window, 159 keV ± 10%. The projection data acquired for 30 min were reconstructed on a Xeleris workstation (GE Healthcare). The ordered-subset expectation maximization method (iterations, three; subset, ten) and a Butterworth filter (critical frequency, 0.5; power, 10.0) were applied to the SPECT images. Both data with and without attenuation correction were generated (no correction [NC]; attenuation correction [AC]). Scatter correction was not used.

### Specific binding ratio index calculation and cerebrospinal fluid-mask processing

We used a commercially available software package for VOI based analysis: DaTView (AZE Co., Ltd., Tokyo, Japan). This software enables the semi-automatic calculation of the SBR index based on the Southampton method and mounts a function of the CSF-mask algorithm.

The CSF-mask algorithm is a threshold process that eliminates low counts caused by brain atrophy or ventricular dilatation. In the CSF-mask algorithm, the threshold is defined as follows:

$$ \mathrm{Threshold}=\overset{\sim }{x}-\sigma \times k $$

where $\overset{\sim }{x}$ and *σ* are the median and standard deviation, respectively, of the background histogram with a Gaussian fit normal distribution and *k* is a coefficient. Previous reports informed our use of 1.0 as the *k* value [8, 10].

The SBR with the CSF-mask algorithm is calculated according to Southampton method as follows:

$$ \mathrm{SBR}=\frac{\frac{{\mathrm{Ctc}}_{\mathrm{VOI}}}{C_{cr}}-{Vc}_{VOI}}{Vs} $$

Where *V*_s_ is the striatal volume (assumed to be 11.2 ml); Ctc_VOI_ represents the total counts in the striatal VOI, excluding the threshold value; C_cr_ is the count concentration in the reference background, excluding the threshold value; and Vc_VOI_ is the volume of the striatal VOI, excluding the threshold volume.

**Fig. 2:**
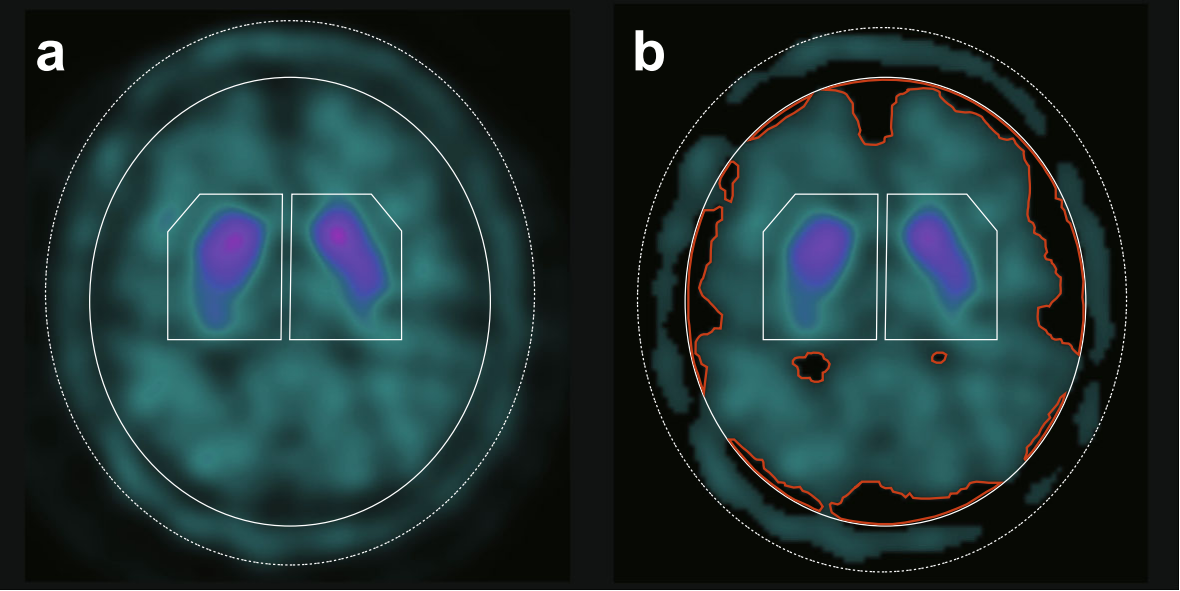
VOI settings with and without the CSF-mask algorithm in a DLB case. **a** The VOI setting without the CSF-mask algorithm and **b** the VOI setting with the CSF-mask algorithm, where CSF-low-count areas are eliminated (regions encompassed by the red line). SBR indices calculated without the CSF-mask algorithm were 4.77 (R) and 4.72 (L) (**a**), while those calculated with the CSF-mask were 3.51 (R) and 3.50 (L) (**b**). The subtle diffuse reduction of tracer uptake in the bilateral striatum is correctly evaluated using the CSF-mask algorithm. *VOI* volume of interest, *CSF* cerebrospinal fluid, *DLB* dementia with Lewy bodies, *SBR* specific binding ratio, *R* right, *L* left

We calculated the SBR indices with and without CSF-mask correction according to the method described above. Figure 2 shows the VOI template as well as the CSF-mask-algorithm-corrected image without the CSF-low-count areas (regions encompassed by the red line).

### Grading the extent of ventricular dilatation by calculating the Evans index

We assessed ventricular size using the EI, which is usually used to diagnose normal pressure hydrocephalus [16]. The EI is defined as the maximal width of the frontal horns of the lateral ventricles divided by the maximal internal diameter of the skull at the same level in axial MR or CT images. In this study, the EI was measured manually using CT (*n* = 119) or MR (*n* = 156) images obtained within 6 months of the DAT SPECT examination. The calculated EIs informed the delimitation of the enrolled cases into three approximately equally sized groups: low EI, middle EI, and high EI.

### Statistical analysis

The Fisher’s exact test or *t* test was used for comparisons of age and sex distribution. The Mann-Whitney *U* test was used to compare the SBR indices between the PS and NPS groups. A receiver-operating characteristics (ROC) analysis was performed to evaluate the area under the curve (AUC). The DeLong method was used to examine the difference between the two AUCs [17]. The sensitivity, specificity, positive predictive value (PPV), negative predictive value (NPV), and accuracy of the SBR indices were calculated using the optimal cut-off values determined based on the ROC curves. Differences with *p* values of < 0.05 (two-sided) were considered to be statistically significant.

The Fisher’s exact test, the *t* test, and the Mann-Whitney *U* test were performed using SPSS software (version 25; SPSS Inc., Chicago, IL). The ROC analysis was performed using the statistical package R (version 3.2.2; available as a free download from http://www.r-project.org).

## Results

### Comparison between the diagnostic performances of the specific binding ratio indices with and without the cerebrospinal fluid-mask algorithm (analysis 1)

**Fig. 3:**
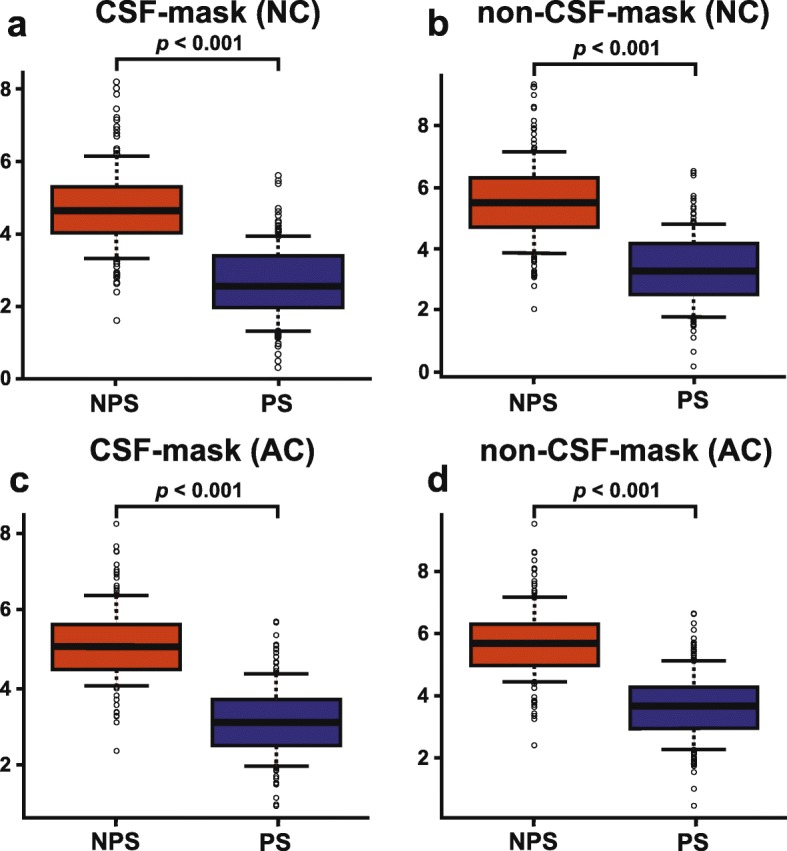
Box-and-whisker plots of the SBRs with CSF-mask in non-attenuation corrected images (**a**), without CSF-mask in non-attenuation corrected images (**b**), with CSF-mask in attenuation-corrected images (**c**), and without CSF-mask in attenuation-corrected images (**d**)

Table 1 shows the characteristics of the included patients for analysis 1. No significant differences were observed with respect to age or the sex ratio between the PS and NPS groups. Figure 3 shows the box-and-whisker plots of the SBRs with and without the CSF-mask algorithm. The mean SBRs with and without the CSF-mask algorithm of patients with NPS were significantly higher than that of patients with PS (4.72 ± 1.18 vs. 2.62 ± 1.02, *p* < 0.001, Fig. 3a; 5.54 ± 1.34 vs. 3.32 ± 1.18, *p* < 0.001, Fig. 3b; 5.09 ± 0.97 vs. 3.10 ± 0.96, *p* < 0.001, Fig. 3c; 5.72 ± 1.13 vs. 3.68 ± 1.11, *p* < 0.001, Fig. 3d; respectively).

**Table 1:** Patient characteristics (analysis 1)

	All	PS	NPS	*p* value
Number of cases	321	163	158	–
Age (y, mean ± SD)	69 ± 12.7	70 ± 10.1	68 ± 14.9	0.09^a^
Men/women (*N*)	176/145	98/65	78/80	0.06^b^

No significant differences were observed between the PS and NPS groups with respect to age or the sex ratio

*PS* parkinsonian syndromes, *NPS* non-parkinsonian syndromes, *SD* standard deviation, *y* years

^a^*t* test

^b^Fisher’s exact test

**Fig. 4:**
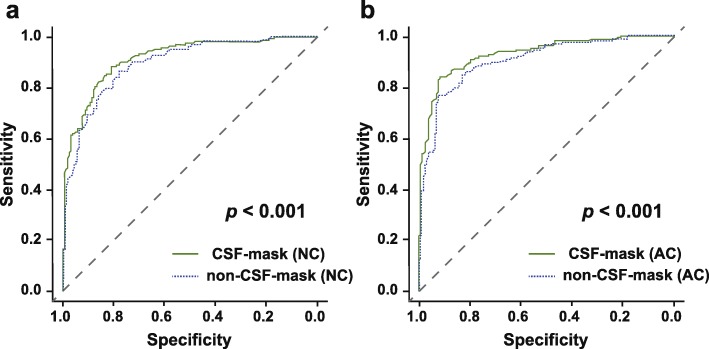
ROC analysis of SBRs with and without the CSF-mask algorithm. The AUCs for the SBRs with and without the CSF-mask algorithm were 0.917 (95% CI 0.887–0.947) and 0.895 (95% CI: 0.861–0.929) in NC (**a**), and 0.930 (95% CI 0.902–0.957) and 0.903 (95% CI: 0.870–0.936) in AC (**b**), respectively. *AC* attenuation correction, *CSF* cerebrospinal fluid, *NC* no correction, *ROC* receiver-operating characteristics, *SBR* specific binding ratio, *AUC* area under the curve, *CI* confidence interval

Figure 4 shows the results of the ROC analyses. The diagnostic performance of the SBR with the CSF-mask algorithm was significantly higher than that of the SBR without the CSF-mask algorithm (NC, AUC = 0.917 [95% confidence interval (CI) 0.887–0.947] vs. 0.895 [95% CI 0.861–0.929], *p* < 0.001, Fig. 4a; AC, AUC = 0.930 [95% CI 0.902–0.957] vs. 0.903 [95% CI 0.870–0.936], *p* < 0.001, Fig. 4b).

Table 2 provides a summary of the sensitivity, specificity, PPV, NPV, and accuracy of the SBRs with and without the CSF-mask algorithm. The cut-off values for the SBRs with and without the CSF-mask algorithm were 3.80 and 4.58 in NC, and 3.97 and 4.78 in AC, respectively.

**Table 2:** Sensitivity, specificity, PPV, NPV, and accuracy of the SBRs with and without the CSF-mask algorithm

	Sensitivity (%)	Specificity (%)	PPV (%)	NPV (%)	Accuracy (%)
Non-CSF-mask (NC)	86.5 (141/163)	77.8 (123/158)	80.1 (141/176)	84.8 (123/145)	82.2 (264/321)
CSF-mask (NC)	88.3 (144/163)	81.0 (128/158)	82.8 (144/174)	87.1 (128/147)	84.7 (272/321)
Non-CSF-mask (AC)	84.7 (138/163)	82.9 (131/158)	83.6 (138/165)	84.0 (131/156)	83.8 (269/321)
CSF-mask (AC)	84.0 (137/163)	91.8 (145/158)	91.3 (137/150)	84.8 (145/171)	87.9 (282/321)

*PPV* positive predictive value, *NPV* negative predictive value, *CSF* cerebrospinal fluid, *SBR* specific binding ratio, *NC* no correction, *AC* attenuation correction

### Impact of the extent of ventricular dilatation on the cerebrospinal fluid-mask algorithm (analysis 2)

**Fig. 5:**
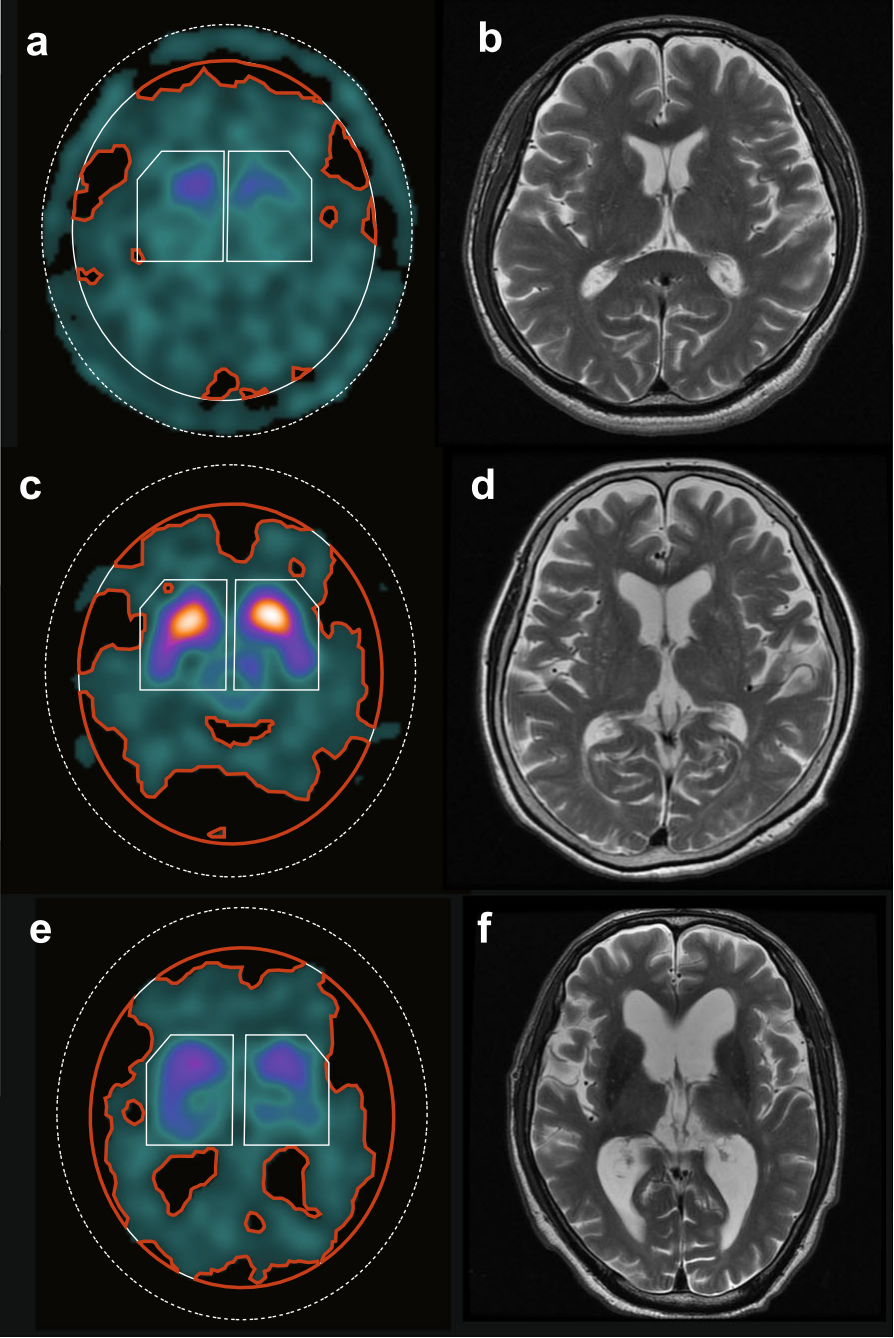
DAT SPECT (**a**, **c**, **e**) and MRI (**b**, **d**, **f**) images of representative cases of the low, middle, and high EI groups. **a** and **b** A resprentative case of the low EI group (age 66, male, EI = 0.217). **c** and **d** A representative case of the middle EI group (age 75, female, EI = 0.279). **e** and **f** A representative case of the high EI group (age 75, male, EI = 0.431)

**Fig. 6:**
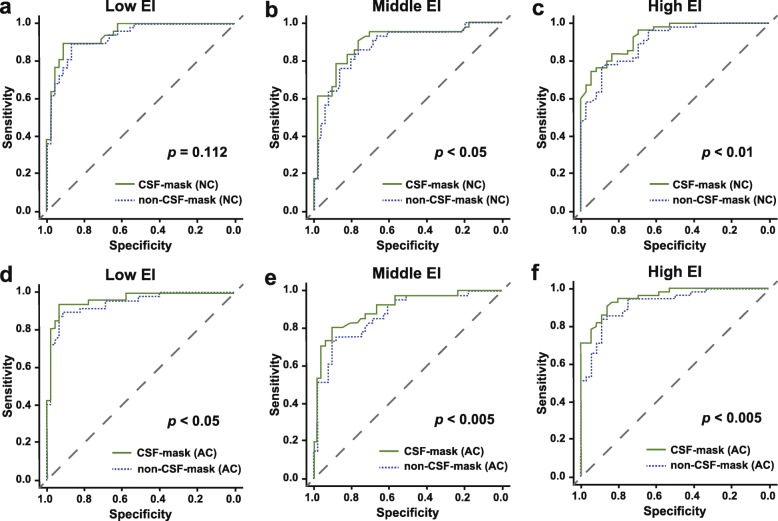
ROC analyses of the SBRs with and without the CSF-mask algorithm in the low, middle, and high EI groups. **a** The AUCs for the SBRs with and without the CSF-mask algorithm were 0.942 (95% CI 0.898–0.986) and 0.927 (95% CI 0.877–0.978), respectively, in the low EI group (NC). **b** The AUCs for the SBRs with and without the CSF-mask algorithm were 0.894 (95% CI 0.825–0.963) and 0.872 (95% CI 0.798–0.947), respectively, in the middle EI group (NC). **c** The AUCs for the SBRs with and without the CSF-mask algorithm were 0.931 (95% CI 0.883–0.978) and 0.900 (95% CI 0.840–0.961), respectively, in the high EI group (NC). **d** The AUCs for the SBRs with and without the CSF-mask algorithm were 0.961 (95% CI 0.924–0.998) and 0.942 (95% CI 0.895–0.988), respectively, in the low EI group (AC). **e** The AUCs for the SBRs with and without the CSF-mask algorithm were 0.905 (95% CI 0.843–0.968) and 0.872 (95% CI 0.800–0.944), respectively, in the middle EI group (AC). **f** The AUCs for the SBRs with and without the CSF-mask algorithm were 0.954 (95% CI 0.917–0.991) and 0.917 (95% CI 0.862–0.973), respectively, in the high EI group (AC). *AC* attenuation correction, *EI* Evans index, *NC* no correction, *ROC* receiver-operating characteristics, *SBR* specific binding ratio, *CSF* cerebrospinal fluid, *AUC* area under the curve, *CI* confidence interval

Table 3 shows the characteristics of the included patients for analysis 2. The mean EIs of the low, middle, and high EI groups were 0.248 (0.215–0.265, *n* = 92), 0.279 (0.265–0.295, *n* = 92), and 0.325 (0.296–0.431, *n* = 91), respectively. Figure 5 presents representative cases of each EI group. Figure 6 presents the results of the ROC analyses. When not corrected for attenuation, in the low EI group, no significant difference was observed between the AUC of the CSF-mask and that of the non-CSF-mask (AUC, 0.942 [95% CI 0.898–0.986] vs. 0.927 [95% CI 0.877–0.978], respectively; *p* = 0.11; Fig. 6a). In the middle and high EI groups, the CSF-mask performed better than the non-CSF-mask (middle EI group, AUC = 0.894 [95% CI 0.825–0.963] vs. 0.872 [95% CI 0.798–0.947], *p* < 0.05, Fig. 6b; high EI group, AUC = 0.931 [95% CI 0.883–0.978] vs. 0.900 [95% CI 0.840–0.961], *p* < 0.01, Fig. 6c). When corrected for attenuation, significant differences in the AUC were observed in all three EI groups (low EI, AUC = 0.961 [95% CI 0.924–0.998] vs. 0.942 [95% CI 0.895–0.988], *p* < 0.05, Fig. 6d; middle EI, AUC = 0.905 [95% CI 0.843–0.968] vs. 0.872 [95% CI 0.800–0.944], *p* < 0.005, Fig. 6e; high EI, AUC = 0.954 [95% CI 0.917–0.991] vs. 0.917 [95% CI 0.862–0.973], *p* < 0.005, Fig. 6f).

**Table 3:** Patient characteristics (analysis 2)

	All	PS	NPS	*p* value
Low EI				
Number of cases	92	47	45	–
Age (y, mean ± SD)	63 ± 15.8	66 ± 11.7	60 ± 18.8	0.04^a^
Men/women (*N*)	43/49	25/22	18/27	0.22^b^
Middle EI				
Number of cases	92	41	51	–
Age (y, mean ± SD)	70 ± 9.8	70 ± 9.2	70 ± 10.3	0.97^a^
Men/women (*N*)	43/49	20/21	23/28	0.83^b^
High EI				
Number of cases	91	56	36	–
Age (y, mean ± SD)	73 ± 7.5	74 ± 7.0	72 ± 8.2	0.20^a^
Men/women (*N*)	64/27	41/14	23/13	0.35^b^

In the low EI group, there was significant difference between the PS and NPS groups with respect to age

*PS* parkinsonian syndromes, *NPS* non-parkinsonian syndromes, *SD* standard deviation, *y* years

^a^*t* test

^b^Fisher’s exact test

## Discussion

The CSF-mask algorithm has been developed to reduce the influence of CSF-low-counts [8]. In this algorithm, threshold process that removes the CSF-low-counts within the reference VOI is applied. Mizumura et al. demonstrated the correctness of this threshold method by comparing it to an MRI-based-mask method that removes CSF-low-counts using MRI images [8]. They proved that the intraclass correlation coefficient indicated high correlation among the SBRs of the MRI-mask and threshold methods, regardless of the reconstruction correction. In a phantom study, Furuta et al. demonstrated that the CSF-mask algorithm significantly eliminated ventricular effects to derive the accurate SBR index [10]. However, they did not assess how much improvement in the diagnostic accuracy using a clinical dataset. To the best of our knowledge, this is the first study to evaluate the effect of the CSF-mask on SBR accuracy in informing the diagnosis of PS using a clinical dataset.

Our overall analysis demonstrated that the diagnostic accuracy of the SBR with the CSF-mask algorithm was higher than that of the SBR without it, revealing the value of routinely using the CSF-mask algorithm when calculating the SBR index with the Southampton method in the diagnosis of PS. The ROC analyses indicate that cut-off values of 3.80 in NC and 3.97 in AC may differentiate PS from NPS when using the SBR with the CSF-mask algorithm; without the CSF-mask algorithm, the recommended cut-off values for the SBR are 4.58 in NC and 4.78 in AC, which are almost the same values as those previously proposed by Tossici-Bolt et al. [7]. The CSF-masked SBR tends to be a lower value than the non-masked one as shown in Fig. 3. We think this is caused by the fact that the excluded CSF region tends to be present in the background VOI more than the striatal VOI, leading the value of the denominator of the equation for finding SBR index become relatively high. As a result, the CSF-masked SBR seems to have a low value compared the non-masked one.

Our study also demonstrated that the effect of the CSF-mask algorithm tends to improve the diagnostic performance in cases with higher EI values. This result confirmed our hypothesis: the stronger the extent of brain atrophy, the more effective the CSF-mask algorithm. Because brain atrophy or ventricular dilatation may occur even early in the course of cognitive decline in patients with PD [11], our results suggest that the CSF-mask algorithm should be used in SBR index calculations for all patients suspected of PD.

The present study is subject to some limitations. First, the diagnoses of PS and NPS were based on clinical diagnoses; thus, we did not use a pathological diagnosis. Although this may have influenced our results, it is difficult to perform DAT SPECT and pathological examination at the same time, and the pathological diagnosis can change with disease progression [18]. It should be also noted that DAT SPECT results are included as indicative biomarkers in the diagnostic criteria of DLB. Second, this was a single-center study; institution-specific factors may therefore limit generalizability. Hence, a multi-center study on the efficacy of employing the CSF-mask to improve the accuracy of SBR-informed diagnoses of PS is warranted. Third, the CSF-mask algorithm cannot be generalized to other VOI settings, for example those provided by DaTQUANT (GE Healthcare, Little Chalfont, UK). Forth, brain atrophy with aging might had an influence on the result of the low EI group because significant difference was observed between the NPS and PS group with respect to age.

## Conclusion

For PS, the diagnostic performance of the SBR index was enhanced by the CSF-mask algorithm, especially in cases with ventricular dilatation.

## Data Availability

All data generated or analyzed during this study are included in this published article.

## Abbreviations

AC: Attenuation correction
AUC: Area under the curve
CSF: Cerebrospinal fluid
DAT: Dopamine transporter
DLB: Dementia with Lewy bodies
EI: Evans index
MSA: Multiple system atrophy
NC: No correction
NPS: Non-parkinsonian syndrome
NPV: Negative predictive value
PD: Parkinson’s disease
PPV: Positive predictive value
PS: Parkinsonian syndrome
ROC: Receiver-operating characteristics
SBR: Specific binding ratio
SPECT: Single-photon emission computed tomography
VOI: Volume of interest
